# Antibacterial, antibiofilm, and anticancer activity of silver-nanoparticles synthesized from the cell-filtrate of *Streptomyces enissocaesilis*

**DOI:** 10.1186/s12896-024-00833-w

**Published:** 2024-02-06

**Authors:** Mohamed T. Shaaban, Briksam S. Mohamed, Muhammad Zayed, Sabha M. El-Sabbagh

**Affiliations:** Botany and Microbiology Department, Faculty of Science, Menoufia University, Shebin El-Kom, Egypt

**Keywords:** Microbial nanotechnology, Nanoparticle synthesis, Silver nanoparticles, Antibacterial activity, Anticancer activity, Biofilm inhibition

## Abstract

Silver nanoparticles (Ag-NPs) have a unique mode of action as antibacterial agents in addition to their anticancer and antioxidant properties. In this study, microbial nanotechnology is employed to synthesize Ag-NPs using the cell filtrate of *Streptomyces enissocaesilis* BS1. The synthesized Ag-NPs are confirmed by ultraviolet-visible (UV-Vis), Fourier transform infrared (FT-IR), X-ray diffraction (XRD), energy dispersive X-ray spectroscopy (EDX), scanning electron microscopy (SEM), and transmission electron microscopy (TEM). Also, the effects of different factors on Ag-NPs synthesis were evaluated to set the optimum synthesis conditions. Also, the antibacterial, antibiofilm, and anticancer activity of Ag-NPs was assessed. The X-ray diffraction (XRD) analysis confirmed the crystalline nature of the sample and validated that the crystal structure under consideration is a face-centered cubic (FCC) pattern. The TEM examination displayed the spherical particles of the Ag-NPs and their average size, which is 32.2 nm. Fourier transform infrared spectroscopy (FTIR) revealed significant changes in functionality after silver nanoparticle dispersion, which could be attributed to the potency of the cell filtrate of *Streptomyces enissocaesilis BS1* to act as both a reducing agent and a capping agent. The bioactivity tests showed that our synthesized Ag-NPs exhibited remarkable antibacterial activity against different pathogenic strains. Also, when the preformed biofilms of *Pseudomonas aeruginosa* ATCC 9027*, Salmonella typhi* ATCC 12023*, Escherichia coli* ATCC 8739*,* and *Staphylococcus aureus* ATCC 6598 were exposed to Ag NPs 50 mg/ml for 24 hours, the biofilm biomass was reduced by 10.7, 34.6, 34.75, and 39.08%, respectively. Furthermore, the Ag-NPs showed in vitro cancer-specific sensitivity against human breast cancer MCF-7 cell lines and colon cancer cell line Caco-2, and the IC_50_ was 0.160 mg/mL and 0.156 mg/mL, respectively. The results of this study prove the ease and efficiency of the synthesis of Ag-NPs using actinomycetes and demonstrate the significant potential of these Ag-NPs as anticancer and antibacterial agents.

**Supplementary Information:**

The online version contains supplementary material available at 10.1186/s12896-024-00833-w.

## Introduction

Antimicrobial resistance (AMR) and cancer pose a global health hazard since they are the primary causes of mortality, and their prevalence continues to rise every year. The deaths associated with bacterial AMR reached 3.62–6.57 million in 2019. The six leading bacterial pathogens (*S. aureus, P. aeruginosa, E. coli, K. pneumoniae, A. baumannii*, and *Strep. pneumoniae*) caused 3.57 million deaths associated with resistance. The methicillin-resistant *S. aureus* (MRSA) alone caused more than 100.000 deaths attributable to AMR in 2019 [1–3]**.** Therefore, the World Health Organization (WHO) recommends that researchers work on developing new antimicrobials to combat the growing health problem of AMR [4, 5]**.**

In the context of developing new antibacterial agents, silver nanoparticles (Ag-NPs) have received considerable attention due to their numerous applications. For example, a new class of silver-based antibiotics has been developed to combat antimicrobial resistance (AMR) based on their unique mode of action as antibacterial agents [6]. It is reported that Ag-NPs promote cell death through the leakage of macromolecules and enzymes. Also, Ag-NPs can destroy the permeability of the bacterial membranes, destroying their structure and resulting in pore formation [7, 8]**.** Moreover, the fact that malignant cells divide in an uncontrolled manner, which ultimately leads to death in both sexes, blew up an urgent need for treatments that are not only safe, inexpensive, and effective but also have fewer negative effects on normal cells. Silver nanoparticles are currently demonstrating activity against a wide range of cancer cell lines, including A549, MCF-7, and caco-2, in addition to other types of cancer cell lines [9, 10]. This killing efficiency goes back to the small particle size, which facilitates its membrane penetration [11]. Furthermore, Ag-NPs have recently been discovered to be useful as anti-tumors due to their cytotoxic, antioxidant, and antidiabetic properties, in addition to augmenting the immunogenicity of vaccines and promoting wound and bone healing [12].

Nanoparticles can be synthesized chemically, physically or by using biological ways [13]. There has always been an obvious interest in controlling the size, shape, and surrounding medium of nanoparticles by all those approaches due to the enormous influence of their size and shape on the electrical and optical properties [14]. Both chemical and physical ways have drawbacks, such as the utilization of harmful chemicals, high energy requirements, low output, and toxic byproducts [15, 16]. Alternate methods for synthesizing such materials with the most minor adverse effects on the environment and human health are highly needed to overcome the drawbacks of other methods and give the utmost yield and purity [17]. Biological resources such as bacteria, actinomycetes, fungi, yeasts, and algae represent safe, high-yield, low-cost alternative sustainable methods for manufacturing nanomaterials [18].

Actinomycetes have the potential to be candidates for intracellular or extracellular nanoparticles formation. The enzymes produced in cellular activities capture the metal ions from the medium and transform these ions into the element metal. In actinomycetes, the intracellular reduction of metal ions can take place either on the surface of mycelia in conjunction with the cytoplasmic membrane or through the transport of metal ions into the microbial cell in the presence of enzymes [19]. Both processes are referred to as “intracellular reduction”, resulting in the formation of nanoparticles as a product. However, extracellular synthesis requires the reductase enzyme [14]. Actinomycetes were found to have further features providing favorable conditions for the biological synthesis of silver nanoparticles because of the negatively charged cell wall. The negative charges in the cell wall allow binding to the positively charged silver ions (Ag^+^) in the silver nitrate metal salt solution. Then, the active molecules and secondary metabolites, including intracellular proteins, cofactors, enzymes such as nitrate reductase, extracellular enzymes, and exopolysaccharides, reduce the silver ions to Ag^0^ [20]. The exopolysaccharides also aid in the distribution of nanoparticles by acting as reducing and stabilizing agents for the synthesis of metal nanoparticles [21, 22].

Moreover, the nanoparticles produced using actinomycetes exhibit exceptional durability and variability in size, along with their biocompatibility, because of their inherent natural capping. Protein capping of Ag-NPs strongly binds silver ions through free amino acid residues, particularly cysteine residues. This protein coating improves the stability of the nanoparticles and prevents their aggregation. A nanoparticle’s coating composition may affect its biocompatibility and operate as an active surface for interactions and/or conjugations with different compounds [23]. Capitalizing on the distinctive features of the biosynthesized nanoparticles derived from the utilized organism, this study employs *Streptomyces enissocaesilis* for the biogenic synthesis of Ag-NPs. The primary focus lies in evaluating the morphological characteristics, antibacterial activity, and cytotoxic effects of the resulting nanoparticles.

## Material and methods

### Sample collection and isolation of actinomycetes

Soil samples from different areas in (latitude of 30.460_N–longitude) El-Menoufia, Egypt, were collected in sterile polythene bags and stored, then processed for isolation and purification. One gram of each sample was shaken in 10 milliliters of sterile deionized water and then left to reconcile for 10 minutes. Serial dilutions ranging from 10^−1^ to 10^−6^ were prepared. Only 0.1 mL of each dilution was inoculated (in triplicate) in a starch nitrate agar (SNA) (soluble starch 20 g, KNO_3_ 2 g, CaCO_3_ 3 g, K_2_HPO_4_ 1 g, MgSO_4_ 0.5 g, NaCl 0.5 g, and agar 20 g / 1 L of distilled water). All plates were incubated at 30 °C for 5 days. Colonies were purified on starch nitrate agar plates using the repeated streak method [24]**.**

### Screening for Ag-NPs synthesis potential

Thirteen actinomycetes isolates were screened for their capacity to make Ag-NPs. The actinomycetes were sub-cultured in starch nitrate broth medium (soluble starch 20 g, KNO_3_ 2 g, CaCO_3_ 3 g, K_2_HPO_4_ 1 g, MgSO_4_ 0.5 g, and NaCl 0.5 g / 1 L of distilled water, pH 8). According to [25, 26], the actinomycetes cell-free culture filtrate was used for Ag-NPs synthesis. The inoculated starch nitrate broth flasks were incubated for 96 hours at 30 °C and 150 rpm, and the media-only flasks served as control. Subsequently, the cultures were centrifuged at 5000 rpm for 15 minutes, and the supernatant (pH 8) was used for the biosynthesis of Ag-NPs. The reaction vessels containing the collected supernatant were mixed with silver nitrate (AgNO_3_), 19:1 respectively, and incubated at 40 °C for 24 hours in the dark. After incubation, the flasks were observed for color change from yellow to brown, which indicates the synthesis of Ag-NPs. A UV-Vis spectrophotometer was then used to validate Ag-NPs synthesis by measuring at a wavelength range of 350 to 700 nm [14]. The strain *Streptomyces enissocaesilis* BS1 was further selected as the most capable strain to produce Ag-NPs.

### Morphological, physiological and molecular identification of *Streptomyces enissocaesilis* BS1

For morphological characterization, the *Streptomyces enissocaesilis* BS1 was grown on SNA (pH 7.5) for 7 days. The colony appearance, pigmentation, and aerial and substrate mycelia colors’ were observed in the medium and recorded. For spore surface morphology, the cover-slip culture method was used. The mycelial spores of *Streptomyces enissocaesilis* BS1 were loaded on the carbon stub, followed by gold sputtering, and imaging was carried out by scanning electron microscopy [27]. Physiological characterization was done to check the growth of the organism at different temperatures ranging from 10 to 50 °C, pH tolerance from pH 5.0 to 9.0, and utilization of carbon sources was done on glucose, sucrose, fructose, lactose, maltose, and starch, beside utilization of nitrogen sources was performed on casein, urea, potassium nitrate, peptone, and ammonium sulphate [23, 28].

Afterward, the strain *Streptomyces enissocaesilis* BS1 was confirmed by PCR amplification of the 16S rRNA gene in the Sequence unit in Macrogen Company, South Korea. The universal primers 27F (5′-AGAGTTTGATCCTGGCTCAG-3′) and 1492R (5′-TACGGYTACCTTGTTACGACTT-3′) [29] were used for PCR amplification and Sanger technique for sequencing. Searches for similarities with nucleotide sequences in the Gen Bank were performed using BLAST (blastn) search, and the sequence was deposited in the GeneBank for accession number. The identified sequences were used to construct the phylogenetic tree [30–32].

### Actinomycetes-mediated reduction of Ag^+^ ions

The strain *Streptomyces enissocaesilis* BS1 was sub-cultured as described previously (In the screening step), and the flasks were observed for color change from yellow to brown, which indicates synthesis of Ag-NPs. Then, to obtain Ag-NPs precipitate from the positive reaction, flasks were centrifuged for 20 minutes at 4 °C and a rotational speed of 12,000 rpm. The collected Ag-NPs precipitates were then washed twice with distilled water and once with methanol as a solvent to remove any attached impurities. Finally, the purified powder was dried in the oven at 50 °C.

### Characterization of Ag-NPs

For confirmation of Ag-NPs formation, a two-beam UV-Vis spectrophotometer was used to measure the absorbance of the reaction mixture at a wavelength range of 300 to 1200 nm [33]. In order to conduct additional tests, an aqueous suspension of silver nanoparticles (Ag-NPs) was created in sterile double-distilled water and then applied to a carbon-coated copper grid. After that, the grid was allowed to dry at room temperature for 15 minutes under infrared light to be photographed using the ZEISS scanning technique for transmission electron microscopy (TEM) [34]. The oven-dried Ag-NPs were loaded onto the stub with carbon tape fixed and coated by sputtering with gold. To determine the morphology of Ag-NPs and characteristics of elemental analysis of Ag-NPs with the help of SEM equipped with energy dispersive X-ray (EDX) [9]. Additionally, the X-ray diffraction (XRD) pattern was obtained by mounting finely powdered silver nanoparticles (Ag-NPs) on a quartz glass slide to create a thin film. Subsequently, the thin film was scanned in the range of 10° ≤ 2ϴ ≤70° (Cu-Kα radiation, wavelength of 1.5406°A, at 30 kV, and 10 mA) using an AXS Bruker X-ray Diffractometer (No. 209450, USA). This process was performed to ascertain the crystalline structure and size of the particle. The Debye-Scherer equation is used to determine the particle size of Ag-NPs. The equation is written as follows:

$$D=\frac{K\lambda}{\beta\ Cos\theta }$$

Where D represents the crystallinity of the Ag-NPs, where K represents the Scherrer constant (0.98), λ represents the X-ray wavelength, β represents the full-width half maximum, and cos θrepresents the observed peak angle.

The acquired diffractogram was compared to the standard card issued by the Joint Committee on Powder Diffraction Standards (JCPDS) with the number 04–0783 [35]. Moreover, the Fourier transform infrared spectrometer (FTIR) (Bruker, USA) was used for documentation of the spectrum of potential bioactive compounds that engage with Ag-NPs [14]. The Ag-NPs dried particle was mixed with potassium bromide (KBr) and studied for the presence of IR bands. The wavelengths at which the spectra were recorded to determine the functional groups are 3500 to 500 cm^−1^ [36].

### Optimization of Ag-NPs synthesis conditions

To optimize the Ag-NPs biosynthesis process, the impact of six variable parameters on the reduction capacity of the supernatant against AgNO_3_ precursor was evaluated by changing one parameter at a time. These parameters included AgNO_3_ concentration (1, 2, 3, 4, or 5 mM), the incubation period (12, 24, and 36 h), temperature (30, 40, 50, 60, and 70 °C), pH (5, 6, 7, 8, and 9), carbon sources (glucose, sucrose, lactose, maltose, starch, and fructose), nitrogen sources (ammonium sulphate, peptone, urea, casein, and potassium nitrate). Finally, flasks were centrifuged at 10000 rpm, and the Ag concentration in the supernatant was measured by ICP. Then, the percentage of reduction was calculated according to the following formula:

$$Reduction\ rate\ \left(\%\right)=\frac{Contol- Sample}{Control}\times 100$$

Where control is the initial silver concentration, and sample refers to the residual silver concentration in the solution, as reported by [37].

### Antibacterial activity and MIC of Ag-NPs

Four bacterial strains (*S. aureus* ATCC 6598, *P. aeruginosa* ATCC 9027, *S. typhi* ATCC 12023, and *E. coli* ATCC 8739) with known antibiotic susceptibility profiles (Table S1) were selected for testing the antibacterial activity of Ag-NPs. The agar well diffusion method [38] was used for testing the antibacterial activity of Ag-NPs against the four bacterial strains. The bacterial suspension of each strain was adjusted at 0.5 McFarland, and a consistent volume of 1 mL of each strain was used to inoculate nutrient agar plates. Distilled water was used as a negative control, and Ampicillin/sulbactam, SAM-20 was used as a positive control. Following that, the well was loaded with 100 μL of 100 mg/mL Ag-NPs, and the plates were incubated at 37 °C for 24 hours. The diameter of inhibition zones surrounding each well was measured.

Furthermore, the microdilution method was performed to assess the minimum inhibitory concentration (MIC); according to [39] In summary, different Ag-NPs concentrations were prepared and added to wells of a 96-well plate, with a total volume of 200 μL, along with the bacterial strains. Then, the 96-well plate was incubated at 37 °C for 24 hours, and the bacterial concentration was detected by measuring the absorbance at O.D  630nm using a Microtiter-plate reader.

The effect of Ag-NPs on the bacterial cell ultra-structure of *S. aureus* ATCC 6598 and *P. aeruginosa* ATCC 9027 was examined using scanning electron microscopy (SEM). After treatment of the bacterial strains with the corresponding MIC of Ag-NPs for 12 hours, the bacterial cells were centrifuged at 3000 rpm for 10 minutes. Then, the cells were washed twice with distilled H_2_O, fixed in 2.5% buffered glutaraldehyde in 0.1 M of phosphate-buffered saline (PBS), pH = 7.4, and then, the bacterial cells were examined using SEM [40].

### Congo red agar (CRA) assay

Following the method described by [39]**,** the CRA assay was used to detect biofilm formation by the strains *S. aureus* ATCC 6598, *P. aeruginosa* ATCC 9027, *S. typhi* ATCC 12023, and *E. coli* ATCC 8739. Brain heart infusion broth (Sigma-Aldrich, St. Louis, MO, USA), 20 g/L of agar powder, and 0.8% Congo red indicator were prepared and autoclaved at 121 °C for 15 minutes. Bacterial strains were streaked on CRA plates; then, the plates were incubated overnight at 37 °C for 24 hours. After incubation, colony color was observed, whereas black colonies with a dry crystalline uniformity indicate biofilm formation.

### Anti-biofilm activity of Ag-NPs

The antibiofilm activity of Ag-NPs against *S. aureus* ATCC 6598, *P. aeruginosa* ATCC 9027, *S. typhi* ATCC 12023, and *E. coli* ATCC 8739 was assessed following the methodology described by [41]. The wells of the microtiter plates were inoculated with 100 μl of bacterial inoculum 10^6^ CFU/mL and then incubated at 37 °C for 24 hours. After that, the remaining bacterial suspensions in the wells of the microtiter plates were discarded, and the plate was left to dry for an hour at 37 °C. Then, Ag-NPs were added to the wells at different concentrations (50, 25, 12.5, 6.25, 3.125, and 1.562 mg/ml) to determine the antibiofilm MIC. The plate was incubated at 37 °C for additional 24 hours, then, the remained solution in the wells was discarded and the wells were washed once with PBS pH 7.2. After drying the wells for 45 minutes, 0.1% crystal violet stain was added to the wells, and the plate was incubated for a further 30 minutes in the dark at 37 °C. Next, the plate was washed once with ethanol (95%, v/v), and then, the reduction of biofilm biomass was detected spectrophotometrically at 570 nm using a microplate ELISA reader (bio-tek, EL × 800). The equation above was employed to calculate the percentage of biofilm biomass elimination [41] as follows:

$$Biofilm\ elimination\%=\frac{OD570\ of\ untreated\ control- OD570\ of\ Ag- NPs- treated\ biofilm}{OD570\ of\ the\ untreated\ control} \times 100$$

### Cell viability assay

The cytotoxic effects of Ag-NPs and percentage of cell viability were determined by colorimetric MTT assay according to the procedure described by [41]. Briefly, the Caco-2 and MCF7 cell lines were trypsinized, washed, and then seeded in 96-well plates at a final density of 10^4^ cells/100 μl, using RPMI-1640 supplemented with 10% FBS with negative (medium with cells but without Ag-NPs) and positive control (Cisplatin), The plates were incubated at 37 °C and 5% CO_2_ for 24 hours. The medium was replaced with FBS-free media containing different concentrations of Ag-NPs at 2-fold serial dilutions (10, 5, 2.5, 1.250, 0.625, 0.3125, 0.156, 0.078125, 0.03906, 0.0195, 0.009 and 0.0048 mg/mL). After 48 hours of incubation at 5% CO_2_ and 37 °C, the wells were washed with PBS. Then, the wells were filled with 50 μL of MTT reagent (3-(4,5-Dimethylthiazol-2-yl)-2,5-diphenyltetrazolium bromide) 0.5 mg/mL PBS, and the plates were then left to sit for 4 hours. The supernatants were thrown away, and the dark blue formazan crystals that had precipitated were dissolved by adding 50 μL of dimethyl sulfoxide 99.9% to each well, followed by stirring. Then, the supernatants were removed, and the number of live cells was determined by measuring the formazan absorbance at 570 nm using a microplate ELISA reader (Bio-Tek, EL800).

The percentage of viability was calculated as follows:

$$Cell\ viability\left(\%\right)=\frac{Mean\ O.D. of\ treated\ cells}{Mean\ O.D. of\ untreated\ cells}\times 100$$

### Statistical analysis

All data are presented as a mean of three independent replicates (mean ± S.E); the significance among different conditions was measured by Tukey’s honest significant difference (Tukey’s HSD, *p* < 0.05) using the Agricola package in R language. Significant differences (*p* < 0.05) between groups at the same phase are shown by different letters.

## Results

### Identification of actinomycetes

**Fig. 1:**
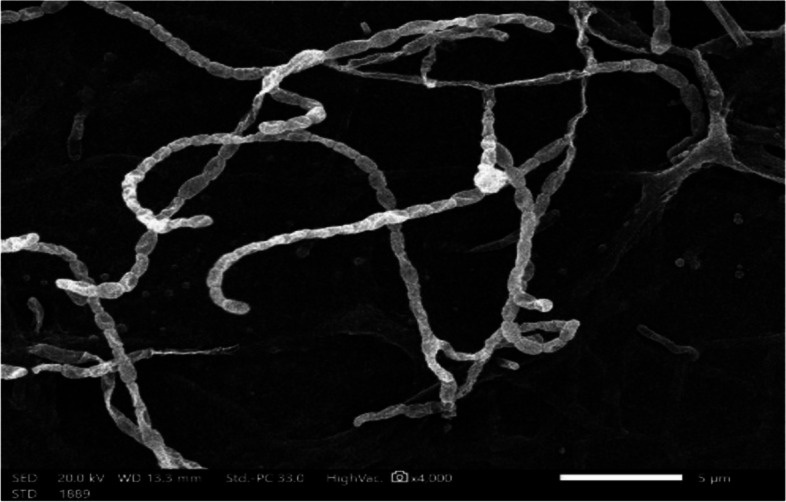
Scanning electron microscope Micrographs showing spore chain morphology

Among the 13 actinomycetes isolates, the strain *Streptomyces enissocaesilis* BS1 was selected as the best Ag-NPs producer. The strain BS1 appeared in SEM micrographs **(**Fig. 1**)** as aerial mycelium-bearing chains of cylindrical spores with smooth surfaces and curves. *Streptomyces enissocaesilis* BS1 isolate showed maximum growth at 30 °C and pH 9. Also, medium growth was observed at 50 °C, pH 8 on SNA medium. *Streptomyces enissocaesilis* BS1 showed a positive response on hydrolysis of starch or casein and a low response on urea hydrolysis. As *Streptomyces enissocaesilis* BS1isolate showed potency on utilization of various nitrogen sources and positive enzyme activities, it can be used in industries and enzyme production.

The 16S rRNA sequencing method was utilized in order to accomplish the molecular characterization of the *Streptomyces enissocaesilis BS1* strain. After the genomic DNA was extracted, primers were used to amplify the region of 16S rRNA that was under investigation. The 16S rRNA partial sequencing that was obtained as 1500 base pairs in length. It was then uploaded to the NCBI database of the GenBank with the accession number OQ132818.1 (https://www.ncbi.nlm.nih.gov/Genbank). After that, a BLAST analysis was performed on this in order to determine whether or not it was closely related to other Streptomyces species. A BLAST analysis revealed that there was a sequence similarity of 99% with the *Streptomyces enissocaesilis* NRRL B-16365 genome. In addition, a phylogenetic tree study of *Streptomyces enissocaesilis BS1* revealed that it has an evolutionary relationship with *Streptomyces enissocaesilis* NRRL B-16365, as can be seen in Fig. S1.

### Actinomycetes-mediated reduction of Ag^+^ ions

The formation of Ag-NPs was detected visually by observing the change of the medium color from yellow to brown, and the color intensity was observed to increase after 24 hours of incubation.

### Characterization of nanoparticles

#### UV-visible (UV-Vis) spectra analysis of Ag-NPs

**Fig. 2:**
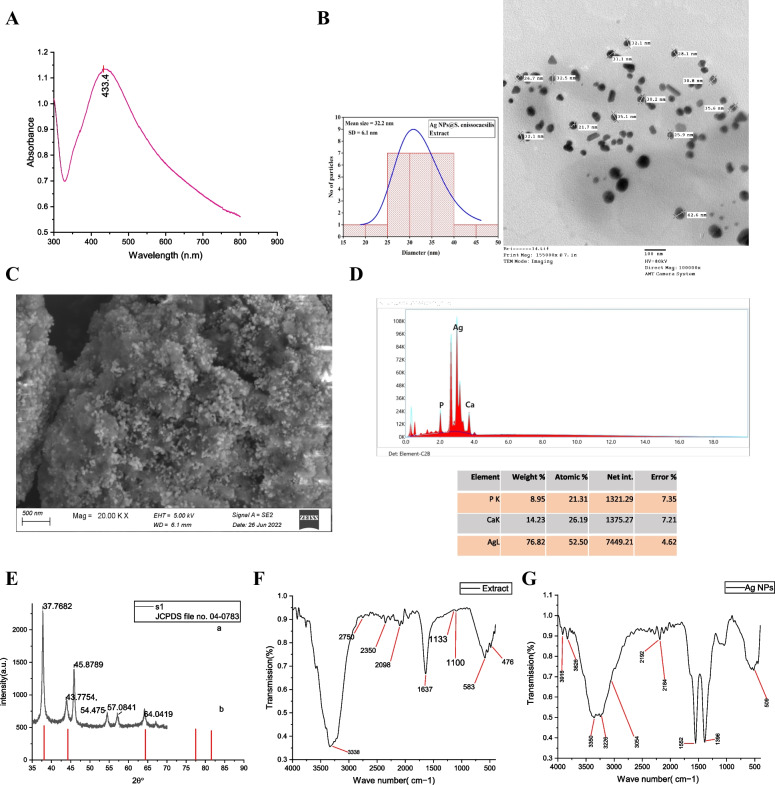
Characterization analyses of the synthesized Ag-NPs; (**A**): The U.V-Vis spectroscopy, (**B**): A TEM micrograph (bar scale 100 nm) and a histogram of the Particle size distribution curve, (**C**): A SEM micrograph (bar scale 500 nm), (**D**): Edax spectrum of Ag-NPs, (**E**): XRD pattern (JCPDS file No. 04–0783), (**F**): FT-IR spectra of the cell-free filtrate of *Streptomyces enissocaesilis* BS1, (**G**): FT-IR spectra of Ag-NPs

The UV-Vis spectral analysis of the optical properties of Ag-NPs revealed a well-defined surface Plasmon resonance band that appeared at around 434 nm (Fig. 2A), which is characteristic of Ag-NPs.

### SEM/EDX and TEM analyses

The TEM micrographs (Fig. 2B) confirmed the formation of well-dispersed spherical Ag-NPs with a mean size of 32.2 nm. Earlier studies published by [42] reported similar sizes. The SEM images of Ag-NPs revealed the accumulation of Ag-NPs in clusters (Fig. 2C). The elemental composition analysis by EDX showed the typical absorption of metallic Ag-NPs with the strongest signal at 3.5 keV of the Ag region (Fig. 2D). Weaker signals from phosphorous and calcium atoms were present due to the impurities from the growth media or capping agents attached to the surface of Ag-NPs.

### X-ray diffraction (XRD)

The x-ray diffraction analysis was done to verify that Ag-NPs are crystalline. The XRD spectrum showed the formation of crystalline Ag-NPs. This crystallinity was observed in the XRD pattern peaks from 35^ℴ^ to 90 ^ℴ^ at 2Ɵ. By comparing the whole spectrum with the standard, the presence of characteristic peaks of silver lattice planes at 111, 200, and 220 was observed (Fig. 2E). The corresponding Bragg’s angle values at (2θ) were 37.77, 43.92, and 64.04, which correlated to Bragg’s reflections of metallic Ag-NPs.

### FTIR measurement

The nature of the biomolecules involved in the capping, reduction, and stability of silver metal was examined using FTIR. The FTIR spectra of the supernatant of *Streptomyces enissocaesilis* BS1 culture indicated various functional groups, as shown in (Fig. 2F). The four showed prominent characteristic peaks at 3338 cm^−1^, 2753 cm^−1^, 2350 cm^−1^, 2100 cm^−1^, 1637 cm^−1^, 1094 cm^−1^, and 582 cm^−1^. The Ag-NPs exhibited peaks at 3916 cm^−1^, 3825 cm^−1^, 3349 cm^−1^, 3226 cm^−1^, 2183 cm^−1^, 1551 cm^−1^, 1395 cm^−1^ and 509 cm^−1^. An intense broad peak centered at 3338 cm^−1^ appeared in the spectra of the cell-free supernatant, ascribing the phenomenon of stretching vibrations of the –O-H and N-H groups. Remarkably, the (−C-H) aliphatic band overlapped with the previous band. Moreover, a single peak of amide (NH2- C=O) appeared at 1637 cm^−1^, beside a tiny peak at 1100 cm^−1^ owing to the C-N-like amine. Significant changes appeared after the addition of Ag-NPs, as shown in (Fig. 2G). The (C-H) aliphatic band did not overlap anymore and appeared at 2979 cm^−1^ with increasing broadness. Two separate peaks appeared at 1552 cm^−1^ and 1388 cm^−1^, indicating the existence of (C=O) and (N-H-), respectively. The peak at 509 cm^−1^ is caused by the metal-ligand stretching frequency that might be a result of biomolecules interacting with the Ag-NPs surface.

### Optimization of Ag-NPs synthesis conditions

The synthesis of Ag NPs was studied under various conditions (AgNO_3_ precursor concentration, incubation period, carbon and nitrogen sources, pH levels, and temperature). It was found that 5 mM of AgNO_3_ precursor was the best concentration for the synthesis of Ag-NPs (Fig. S2a). Therefore, this concentration was adjusted in the following experiments. The highest Ag-NPs concentration was detected after 24 hours of incubation; however, extending the incubation period for an additional 12 hours reduced the reduction rate (Fig. S2b). Our experimental findings determined that starch as the most efficient carbon source, followed by fructose and sucrose, respectively, and glucose was the lowest efficient one (Fig. S2c). Potassium nitrate KNO_3_ considerably increased the reduction rate as a nitrogen source (Fig. S2d). The pH optimization tests revealed that the best Ag-NPs synthesis rate was observed at pH 9 (Fig. S2e). Additionally, our findings revealed that increasing the temperature to 60 °C enhanced the reduction rate compared to 30 °C, but increasing the temperature to 70 °C decreased the reduction rate (Fig. S2f).

### Antibacterial and antibiofilm activity of Ag-NPs

**Fig. 3:**
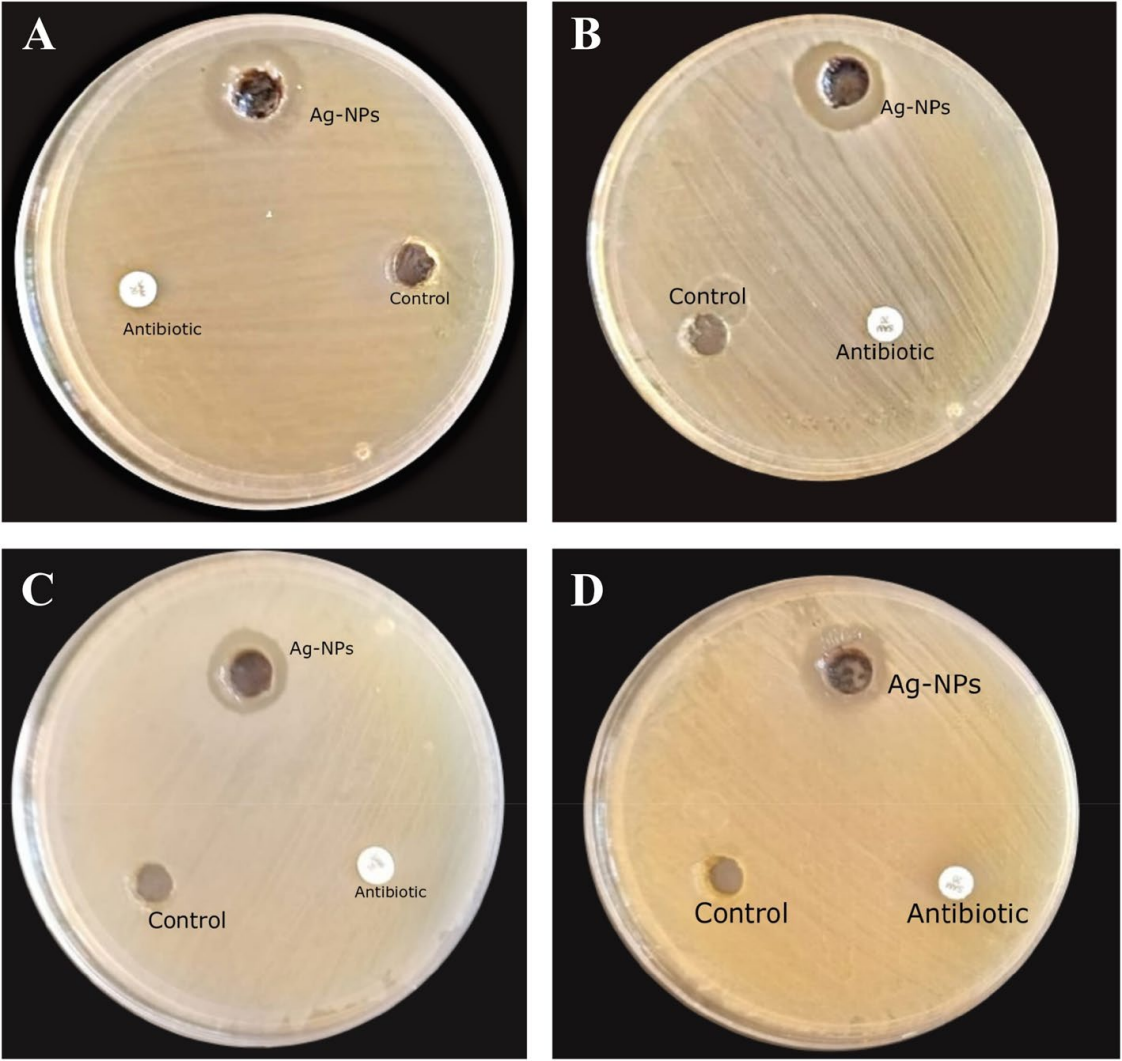
Zone of inhibitions obtained with Ag-Nps compared to positive control (antibiotic) and negative control against the four test pathogenic strains; (**A**): *P. aeruginosa*; (**B**): *S, typhi*; (**C**): *E. coli*; (**D**): *S. aureus*

**Fig. 4:**
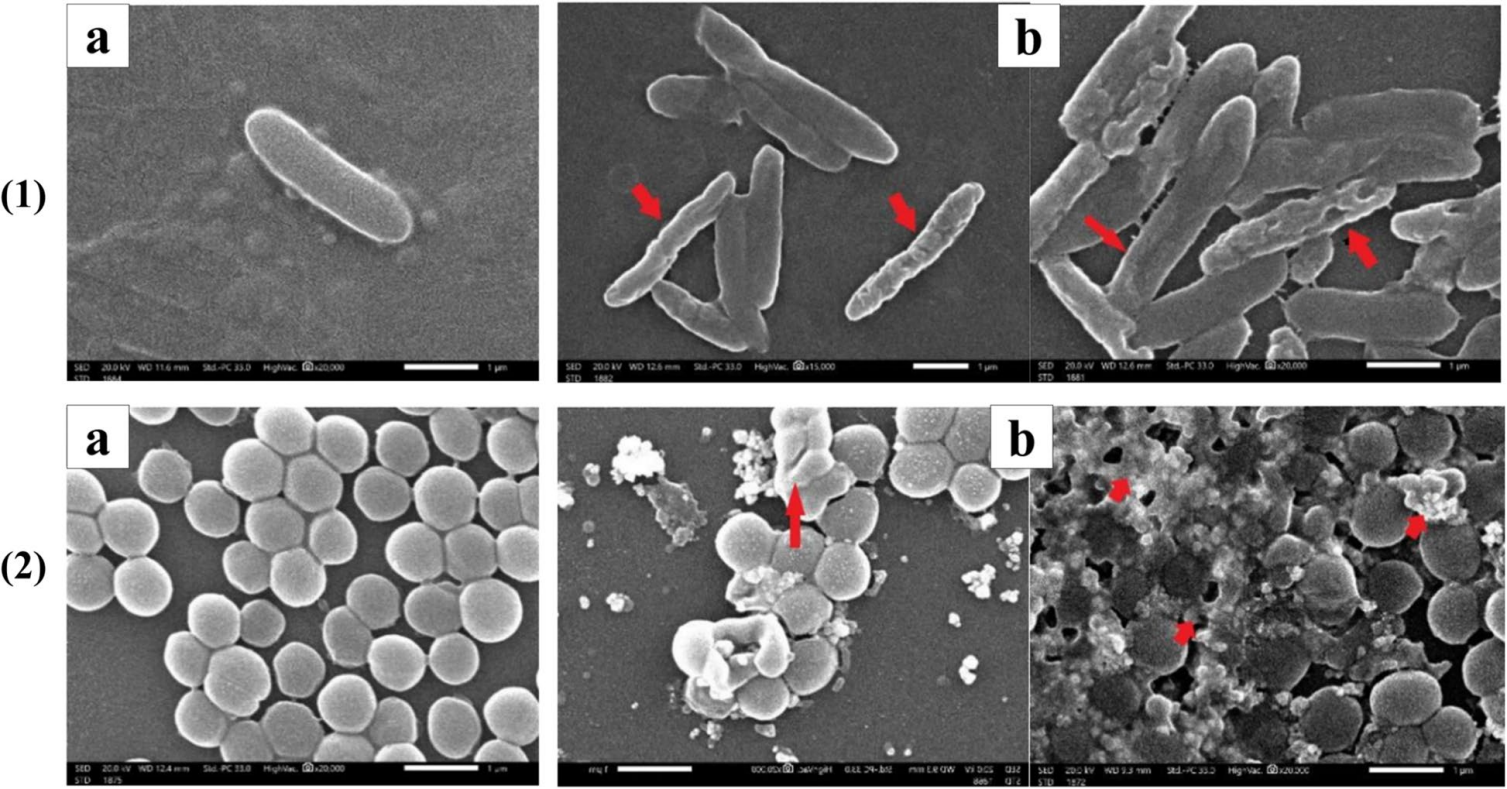
SEM micrograph comparing the ultra-structure of Ag-NPs- treated *P. aeruginosa, S. aureus* (1b, 2b) with the control (1a, 2a). Red arrows point to the deformation of the bacterial cell membrane

The agar well diffusion method was used for preliminary testing of the antibacterial activity of Ag-NPs. The Ag-NPs showed visible inhibition zones against the four tested bacterial strains (*S. aureus* ATCC 6598, *P. aeruginosa* ATCC 9027, *S. typhi* ATCC 12023, and *E. coli* ATCC 8739). The inhibition zones and MIC of Ag-NPs against each bacterial strain are shown in (Table 1, Figs. 3 and S3). Additionally, the effect of Ag-NPs on the cellular ultrastructure of *S. aureus* and *P. aeruginosa* was examined by SEM before and after treatment. The SEM micrographs revealed the control to have normal morphological characteristics (Figs. 4; Panels 1a and 2a), displaying a smooth, undamaged cell membrane. Nevertheless, the cell membrane of Ag-NPs-treated *S. aureus* exhibited signs of impairment, characterized by the emergence of many holes besides completely lysed cells (Fig. 4; Panel 1b). Also, the deformation of the cell membrane of Ag-NPs-treated *P. aeruginosa* appeared as serrated, as shown in (Fig. 4; Panel 2b).

**Table 1:** Antibacterial activities of Ag-NPs

Bacterial strain	Average diameter of inhibition zone (mm)	Ag-NPs MIC (mg/ml)
*S. aureus*	16.6 ± 0.5	0.781 mg/ml
*E. coli*	16.3 ± 0.5	1.56 mg/ml
*S. typhi*	14.5 ± 0.6	0.781 mg/ml
*P. aeruginosa*	16 ± 0.4	0.781 mg/ml

For testing the antibiofilm activity of Ag-NPs, the bacterial strains were first tested for their capability to form biofilm. Using the CRA plates, all of the tested strains showed the ability to form biofilms. These biofilms were detected through the formation of black colonies on the CRA plates (Fig. S4). Afterward, using the microtiter plate method, the antibiofilm activity of different concentrations (ranging from 50 to 1.56 mg/ml) of Ag-NPs was assessed. The Ag-NPs were found to possess a dose-dependent capacity to eliminate the preformed biofilms of *S. aureus* ATCC 6598, *P. aeruginosa* ATCC 9027, *S. typhi* ATCC 12023, and *E. coli* ATCC 8739. As illustrated in (Table 2), 50 mg/ml of Ag-NPs was able to eliminate and reduce the biomass of the preformed biofilms of *P. aeruginosa*, *S. typhi*, *E. coli*, and *S. aureus* by 10.7, 34.6, 34.75, and 39.08%, respectively, after 24 hours of exposure.

**Table 2:** Percentages of biofilm biomass reductions after treatments with different Ag-NPs concentrations

Ag-NPs Conc. (mg/mL)	Bacterial strains			
	*S. typhi*	*P. aeruginosa*	*S. aureus*	*E. coli*
50	34.6% ± 0.018	10.74% ± 0.019	39.08% ±0.03	34.75% ± 0.00
25	28.4 ± 0.054	10.84% ± 0.12	25.67% ± 0.062	33.93% ± 0.05
12.5	28% ± 0.075	9.34% ± 0.05	25.53% ± 0.00	33.77% ± 0.06
6.25	21.3% ± 0.19	9.28% ± 0.09	6.56% ± 0.050	10.98% ± 0.13
3.125	16.26%0.13	7.47% ± 0.013	6.56% ± 0.050	11.8% ± 0.10
1.56	14.26% ± 0.005	1.49% ± 0.001	1.1% ± 0.07	6.06% ± 0.006

### Anticancer activity

**Fig. 5:**
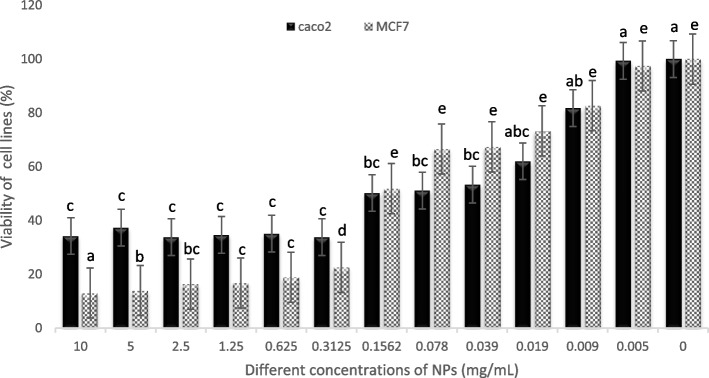
In vitro anticancer activity of Ag-NPs against breast cancer MCF7 cell line and colon cancer CaCO-2 cell line. All values are presented as mean ± SD (*n* = 3). Significant differences (*p* < 0.05) between groups are shown by different letters

A standard MTT test was used to assess the cytotoxicity of Ag-NPs by comparing the viability of the breast cancer MCF7 cell line and colon cancer Caco-2 cell line with the control group (Fig. 5). After 48 hours of exposure to concentrations ranging from 0.0048 to 10 mg/ml (two-fold serial dilutions), The treatment of cancer cells with the various Ag-NPs concentrations yielded a remarkable reduction in viable cells of MCF7, declining from 97.0 to 13% and from 99 to34% in Caco-2. The untreated cells displayed a cell viability rate of 100%, while the standard drug cisplatin exhibited a cell viability rate of 23.45 and 18.23% with MCF7 and Caco-2, respectively. The Ag-NPs indicate a promising anticancer potential with an IC50 value of 0.160 mg/ml and 0.156 mg/ml for MCF7 and Caco-2, correspondingly. Morphological changes, including cell shrinkage and rounding at varying degrees, were noticed in the treated cells compared to untreated, as shown in (Fig. S5).

## Discussions

In a time marked by rising bacterial antimicrobial resistance and elevated mortality rates associated with both AMR [1] and various cancer types [43], it becomes crucial to focus on the development of new antibacterial and anticancer agents. In this context, this study aimed at exploiting the actinomycetes for the synthesis of silver nanoparticles as a potential antimicrobial and antitumor activity. The cell-free filtrate of *Streptomyces enissocaesilis* BS1 was used for the synthesis of silver nanoparticles (Ag-NPs). It is reported that the synthesized nanoparticles using actinomycetes have significant stability and biocompatibility due to the natural capping, which prevents their aggregation [23]. To evaluate the characteristics of the Ag-NPs, several analyses were employed. UV-Vis was used for spectra analysis, and the results agreed with the other published results [44, 45]. The spectral analysis showed the appearance of a well-defined surface Plasmon resonance band at 434 nm (Fig. 2A), which is characteristic of silver nanoparticles.

Moreover, the TEM and SEM examination revealed the formation of spherical Ag-NPs sized from 21.7 nm to 42.6 nm (Fig. 2B, C). Nanoparticles in such shape and size range are considered advantageous since they facilitate cell uptake and do not trigger the immune response due to their relatively large surface area [46]. In accordance with the findings of [47], the EDX spectrum of the biosynthesized Ag-NPs showed the typical absorption of metallic Ag-NPs. Despite the presence of weaker signals due to the impurities, the EDX spectrum analysis confirmed the formation of silver nanoparticles represented by the strong signal at 3.5 keV of Ag region (Fig. 2D). The x-ray diffraction analysis confirmed the formation of crystalline Ag-NPs in the pattern peaks from 35^ℴ^ to 90 ^ℴ^ at 2Ɵ (Fig. 2E). The corresponding Bragg’s angle values at (2θ) were 37.77, 43.92, and 64.04, which correlated to Bragg’s reflections of metallic Ag-NPs. Similar outcomes were also reported by [41], and these findings support the face-centered cubic structure of the Ag-NPs as determined by the Joint Committee on Powder Diffraction Standards (JCPDS file no. 04–0783). In addition, there were unassigned peaks, which might be an indication that the bio-organic phase crystallizes on the nanoparticles’ surfaces.

The FTIR analyses of Ag-NPs indicated an intense broad peak centered at 3338 cm^−1^ appeared in the spectra of the extract, attributed to stretching vibrations of the (−O-H) and N-H groups. Remarkably, the (−C-H) aliphatic band overlapped with the previous band. Moreover, a single peak of amide (NH2- C=O) appeared at 1637 cm^−1^, beside a tiny peak at 1100 cm^−1^ owing to the C-N-like amine. While significant changes appeared after the addition of silver nanoparticles, The (C-H) aliphatic band did not overlap anymore and appeared at 2979 cm^−1^ with increasing broadness. Two separated peaks appeared at 1552 cm^−1^ and 1388 cm^−1^, which indicates the occurrence of (C=O) and (N-H-), respectively. The presence of amide linkages in proteins participating in the bio-reduction of AgNO_3_ can be inferred through the identification of N-H stretch vibrations [48, 49], which have theorized that the presence of reactive N-H and O-H groups renders Ag(I) ions more susceptible to reduction, resulting in the formation of Ag(0).

Prior to evaluating the biological activity of the synthesized Ag-NPs, it was important to optimize the production conditions for an efficient reproducible synthesis process. AgNO_3_ served as a precursor, and the ICP-MS was used for the determination of Ag-NPs synthesis optimal conditions by assessing the reduction rate. As detailed in the results sections and expressed in (Fig. S2), our results indicated that 5 mM of AgNO_3_ yielded the highest reduction rate. Similar findings were attained by [50]. This conclusion aligns with the findings of [51], who observed a correlation between the development of a darker-colored solution and an increase in silver nitrate concentrations from 1:5 mM. It is theorized that this was due to a more efficient conversion (better nucleation) of silver ions into silver nanoparticles (Ag-NPs), with a higher yield of Ag-NPs.

The increased reduction rate was observed at temperatures increasing from 30 to 60 °C, suggesting a higher rate of Ag-NPs synthesis. However, the reduction rate decreased when the temperature rose to 70 °C. The observable decrease in Ag-NPs at 70 °C is thought to be due to denaturation or deactivation of the enzymes (responsible for bio-reduction), which are protein in nature and could be heat sensitive. It is reported that pH can directly affect the size of nanoparticles and the electrical charges of biomolecules, which may affect both their ability to cap and stabilize and the subsequent growth of the nanoparticles [44]. Our results also showed that the optimum pH for Ag-NPs synthesis is pH 9. The Ag-NPs production was not facilitated by the acidic media (pH 5–6). Colors range from colorless to yellow, indicating the influence of pH on the size effect. Furthermore, the solution of Ag-NPs became lighter in color in the acidic media.

It is reported that an organic layer, such as a protein, usually caps the Ag-NPs produced from the reduction of silver by microorganisms. Such reductions are governed by many factors which in turn govern the behavior of Ag NPs [37]. Therefore, it is necessary to select the optimum carbon and nitrogen source. Our results showed that, under optimal conditions, KNO_3_ exhibited the highest reduction rate of AgNO_3_. Comparable findings were obtained by [52] when they employed the green microalga *Scenedesmus obliquus* for the extracellular green synthesis of Ag-NPs, where KNO_3_ demonstrated superior performance in synthesizing relatively small-size Ag-NPs with optimal antimicrobial properties.

The synthesized Ag-NPs were assessed for their antibacterial activity against four important bacterial pathogens, including three of the six leading bacterial pathogens (*S. aureus, E. coli,* and *P. aeruginosa*), causing deaths associated with antibiotic resistance. The Ag-NPs efficiently inhibited the growth of all the tested strains. The SEM micrographs (Fig. 3) revealed caused cell membrane deformation caused by Ag-NPs. This membrane destruction is attributed to the small size and the spherical shape of nanoparticles, which present a larger surface area, facilitating greater surface reactions against target bacteria [42, 53].

Since the bacterial biofilms hinder the resistance and penetration of the antibiotics, the Ag-NPs were tested for their anti-biofilm activity. The results revealed the dismissal of pre-formed biofilm biomass of the four tested strains by 10–39% after 24 hours of exposure to 50 mg/mL Ag-NPs. In the time that antibiotics cannot penetrate the biofilm matrix, the small size of Ag-NPs allowed the particles to cross the matrix and show the observable reduction of the biofilm matrix. Due to its small size, Ag-NPs can more easily reach the sugary layer and contact the bacterial cells, preventing biofilm formation [54].

Cancer has been a leading death cause around the world [43], which creates a situation where new anticancer agents are required. Herein, Ag-NPs were assessed for inhibiting the viability of the breast cancer MCF7 cell line and colon cancer Caco-2 cell line. The IC50 of Ag-NPs for MCF7 and Caco-2 was 0.16 and 0.156 mg/ml, respectively. It is noticed that Ag-NPs caused a dose-dependent reduction in cell viability. Similar results were obtained by [48], and such cytotoxicity of Ag-NPs at low concentrations backs to its small size. Comparable findings published by [10] show that the viability of the Caco-2 cell line is inversely proportional to the concentration of Ag-NPs. The viability of the Caco-2 cell line descended by 98.0, 68.8, 46.5, 28.6, and 4.8% at doses of Ag-NPs ranging from 12.5 to 200 μg/mL, respectively. When the concentration of C-Ag-NPs was 200 μg/mL, the highest drop in cell viability was found to be 4.8%. Ag-NPs have an IC50 value of 0.049 mg/mL. In comparison, a dose-dependent decrease in cell viability was observed after 48 hours of treatment for breast cancer cell lines. In the study, the IC50 of Ag-NPs was found to be 3.043 μL/mL. It was found that a maximal dose of 25 μg/mL was sufficient to achieve a complete cell inhibition of breast cancer cell lines, which was 99%.

## Conclusions

This study introduces synthesized silver nanoparticles (Ag-NPs) as antibacterial and anticancer agents. The small size and the characteristics of the synthesized Ag-NPs make these particles a potential future antibacterial and anticancer therapeutic. This study was limited to the synthesis of Ag-NPs from the cell filtrate of actinomycetes, characterization and evaluation of their antibacterial and antitumor activity. Therefore, it is recommended that future studies on such particles should be extended to include in vivo models and drug safety experiments. This study and similar studies play a role in providing the antibacterial and anticancer drug development pipeline with new active agents. Also, this study orients attention to the green production of nanoparticles using safe, beneficial microorganisms such as actinomycetes, as the use of these microorganisms allows the application of the One Health concept in nanoparticle manufacturing.

### Future prospectives

There is an immediate demand for getting back to nature and producing treatments that are non-toxic, affordable, and effective, with low adverse effects at the same time. All those can be provided by natural sources like plants, actinomycetes, and other microbes. We advocate the possibility of harnessing nanotechnology to be exploited in medicine and the development of anticancer nano-drugs, as well as examining the level of safety over normal cells and investigating the synergistic effect of adding silver nanoparticles to anticancer drugs in order to guarantee that the effectiveness of the treatment will enhance.

### Supplementary Information

**Additional file 1: Table S1.** Antibiotic susceptibility profile of the bacterial strains (Testing, 2019).

**Additional file 2: Fig. S1.** A maximum likelihood phylogenetic tree built using fragments of 16S rDNA from different bacterial strains. The phylogenetic tree is anchored using the homologous sequence of *Escherichia coli* NW_A26 in order to demonstrate the evolutionary relationships between the homologous sequences of *Streptomyces enissocaesilis* BS1 16S rDNA. The iTOL (Interactive Tree of Life) website was utilized to display and visualize the downloaded phylogenetic trees. **Fig. S2.** Effect of different growth factors on the synthesis of silver nanoparticles expressed as reduction rate; (A) precursor concentration; (B) Incubation period; (C) Carbon source; (D) Nitrogen source; (E) pH level; (F) Temperature of incubation. The significance among different conditions was measured by Tukey’s honest significant difference (Tukey’s HSD, *p* < 0.05) using Agricolae package in R language [50, 51]. Identical letters indicate that the difference is not statistically significant. Identical letters indicate that the difference is not statistically significant. **Fig. S3.** The 96-well plate of MIC determination test of Ag-NPs against the bacterial test strains. **Fig. S4.** Biofilm formation test by the four test strains; (A): *P. aeruginosa*; (B): *S. aureus*; (C): S, typhi; (D): *E. coli*. Appearance of black colonies or change of media color from red to black below the growth indicates formation of biofilm, and the unchanged red color of the medium indicates absence of biofilm. **Fig. S5.** Morphological changes in MCF-7 cell line after exposure to Ag-NPs: (A1) untreated cells (B1) treated cells. Moreover, Morphological changes in Caco-2 cell line after exposure to Ag-NPs: (A2) untreated cells, (B2) treated cells.

## Data Availability

No datasets were generated or analysed during the current study.
