# Supplementary material for: Antibacterial, antibiofilm, and anticancer activity of silver-nanoparticles synthesized from the cell-filtrate of Streptomyces enissocaesilis
Source: BMC Biotechnol. 2024 Feb 6;24:8. doi: 10.1186/s12896-024-00833-w (PMC10848522; doi:10.1186/s12896-024-00833-w)
Supplement: Supplementary file 1 — Additional file 1: Table S1. Antibiotic susceptibility profile of the bacterial strains (Testing, 2019). [file 12896_2024_833_MOESM1_ESM.docx]

| **Table S1:** Antibiotic susceptibility profile of the bacterial strains(Testing, 2019). | | | | | | |
| --- | --- | --- | --- | --- | --- | --- |
| **Antibiotic** | **Susceptibility** | **Diameter of inhibition (mm)** | **Bacterial strains** | | | |
|  |  |  | ***S. aureus*** | ***E. coli*** | ***S. typhi*** | ***P. aeruginosa*** |
| **Norflaxicin**  **(10mg)** | R | ≤ 12 | S | R | R | S |
|  | S | ≥ 17 |  |  |  |  |
| **Meropenem**  **(10mg)** | R | ≤ 19 | S | S | S | S |
|  | S | ≥ 23 |  |  |  |  |
| **Amoxicilin + clavulnic**  **(20/10mg)** | R | ≤ 19 | S | R | - | S |
|  | S | ≥ 20 |  |  |  |  |
| **Ampicillin**  **(10 mg)** | R | ≤ 18 | S | S | S | R |
|  | S | ≥ 22 |  |  |  |  |
| **Nalidixic acid**  **(30mg)** | R | ≤ 13 | - | S | S | R |
|  | S | ≥ 19 |  |  |  |  |
| **Chloramphenicol**  **(30mg)** | R | ≤ 12 | S | S | S | R |
|  | S | ≥ 18 |  |  |  |  |
| **ciproflaxcin (5mg)** | R | ≤ 20 | S | R | S | S |
|  | S | ≥ 31 |  |  |  |  |
| **Levoflaxcin**  **(5mg)** | R | ≤ 14 | S | R | S | S |
|  | S | ≥ 22 |  |  |  |  |
| **Tetracyclin**  **(30 mg)** | R | ≤ 11 | S | R | - | R |
|  | S | ≥ 15 |  |  |  |  |
| **Azithromycin**  **(15mg)** | R | ≤ 12 | - | R | - | R |
|  | S | ≥ 13 |  |  |  |  |
| **Doxycycline**  **(30mg)** | R | ≤ 10 | - | S | - | R |
|  | S | ≥ 14 |  |  |  |  |
| **Vancomycin**  **(30mg)** | R | ≤ 14 | S | - | - | - |
|  | S | ≥ 17 |  |  |  |  |
| MAR Index % | | | 0 | 0.54 | 0.14 | 0.54 |

Testing, S. (2019). *Clsi*. https://doi.org/10.1007/978-3-662-48986-4_300418
