# Supplementary material for: Antibacterial, antibiofilm, and anticancer activity of silver-nanoparticles synthesized from the cell-filtrate of Streptomyces enissocaesilis
Source: BMC Biotechnol. 2024 Feb 6;24:8. doi: 10.1186/s12896-024-00833-w (PMC10848522; doi:10.1186/s12896-024-00833-w)
Supplement: Supplementary file 2 — Additional file 2: Fig. S1. A maximum likelihood phylogenetic tree built using fragments of 16S rDNA from different bacterial strains. The phylogenetic tree is anchored using the homologous sequence of Escherichia coli NW_A26 in order to demonstrate the evolutionary relationships between the homologous sequences of Streptomyces enissocaesilis BS1 16S rDNA. The iTOL (Interactive Tree of Life) website was utilized to display and visualize the downloaded phylogenetic trees. Fig. S2. Effect of different growth factors on the synthesis of silver nanoparticles expressed as reduction rate; (A) precursor concentration; (B) Incubation period; (C) Carbon source; (D) Nitrogen source; (E) pH level; (F) Temperature of incubation. The significance among different conditions was measured by Tukey’s honest significant difference (Tukey’s HSD, p < 0.05) using Agricolae package in R language [50, 51]. Identical letters indicate that the difference is not statistically significant. Identical letters indicate that the difference is not statistically significant. Fig. S3. The 96-well plate of MIC determination test of Ag-NPs against the bacterial test strains. Fig. S4. Biofilm formation test by the four test strains; (A): P. aeruginosa; (B): S. aureus; (C): S, typhi; (D): E. coli. Appearance of black colonies or change of media color from red to black below the growth indicates formation of biofilm, and the unchanged red color of the medium indicates absence of biofilm. Fig. S5. Morphological changes in MCF-7 cell line after exposure to Ag-NPs: (A1) untreated cells (B1) treated cells. Moreover, Morphological changes in Caco-2 cell line after exposure to Ag-NPs: (A2) untreated cells, (B2) treated cells. [file 12896_2024_833_MOESM2_ESM.pptx]

## Slide 1
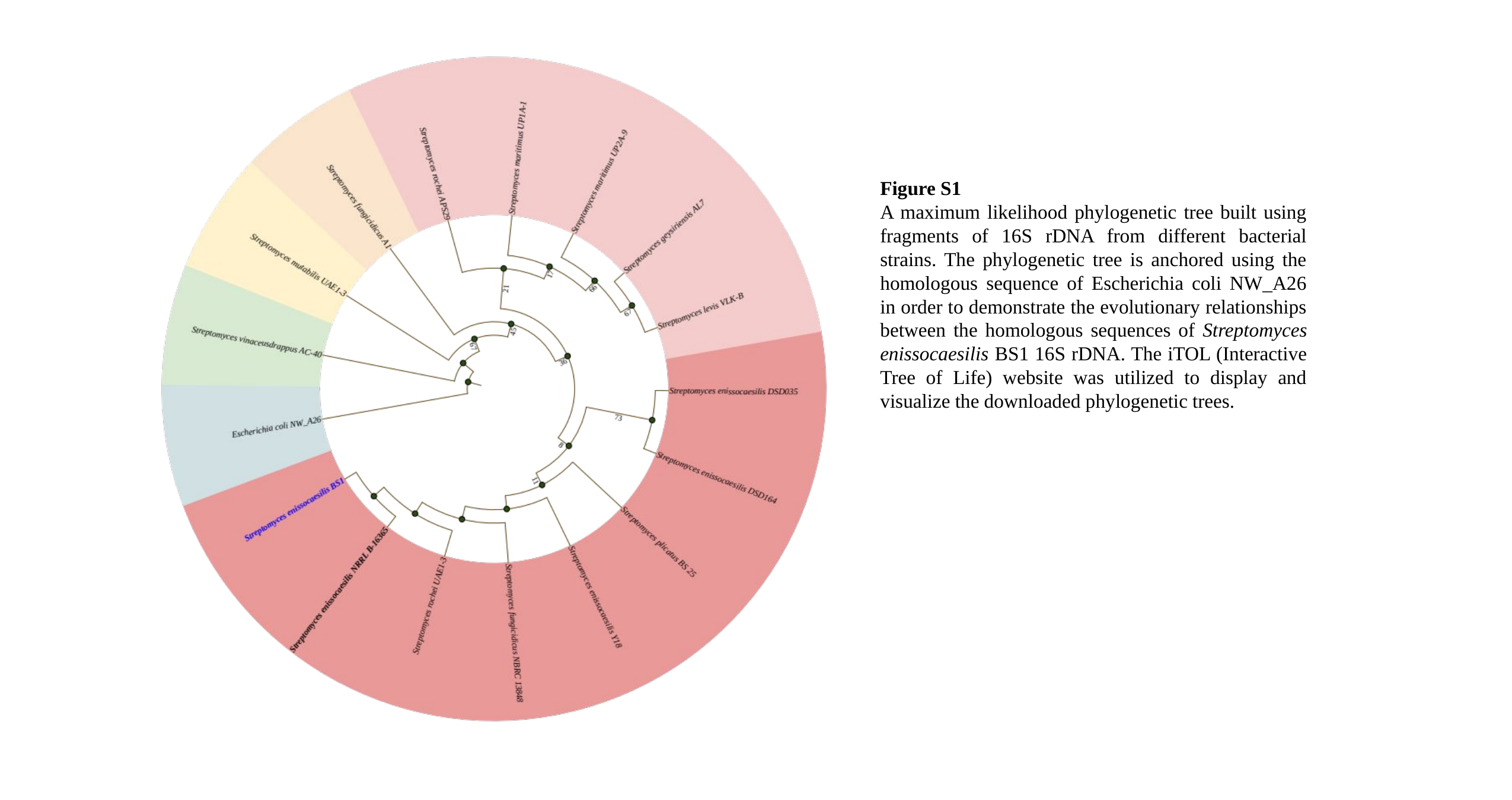

Figure S1
A maximum likelihood phylogenetic tree built using fragments of 16S rDNA from different bacterial strains. The phylogenetic tree is anchored using the homologous sequence of Escherichia coli NW_A26 in order to demonstrate the evolutionary relationships between the homologous sequences of Streptomyces enissocaesilis BS1 16S rDNA. The iTOL (Interactive Tree of Life) website was utilized to display and visualize the downloaded phylogenetic trees.

## Slide 2
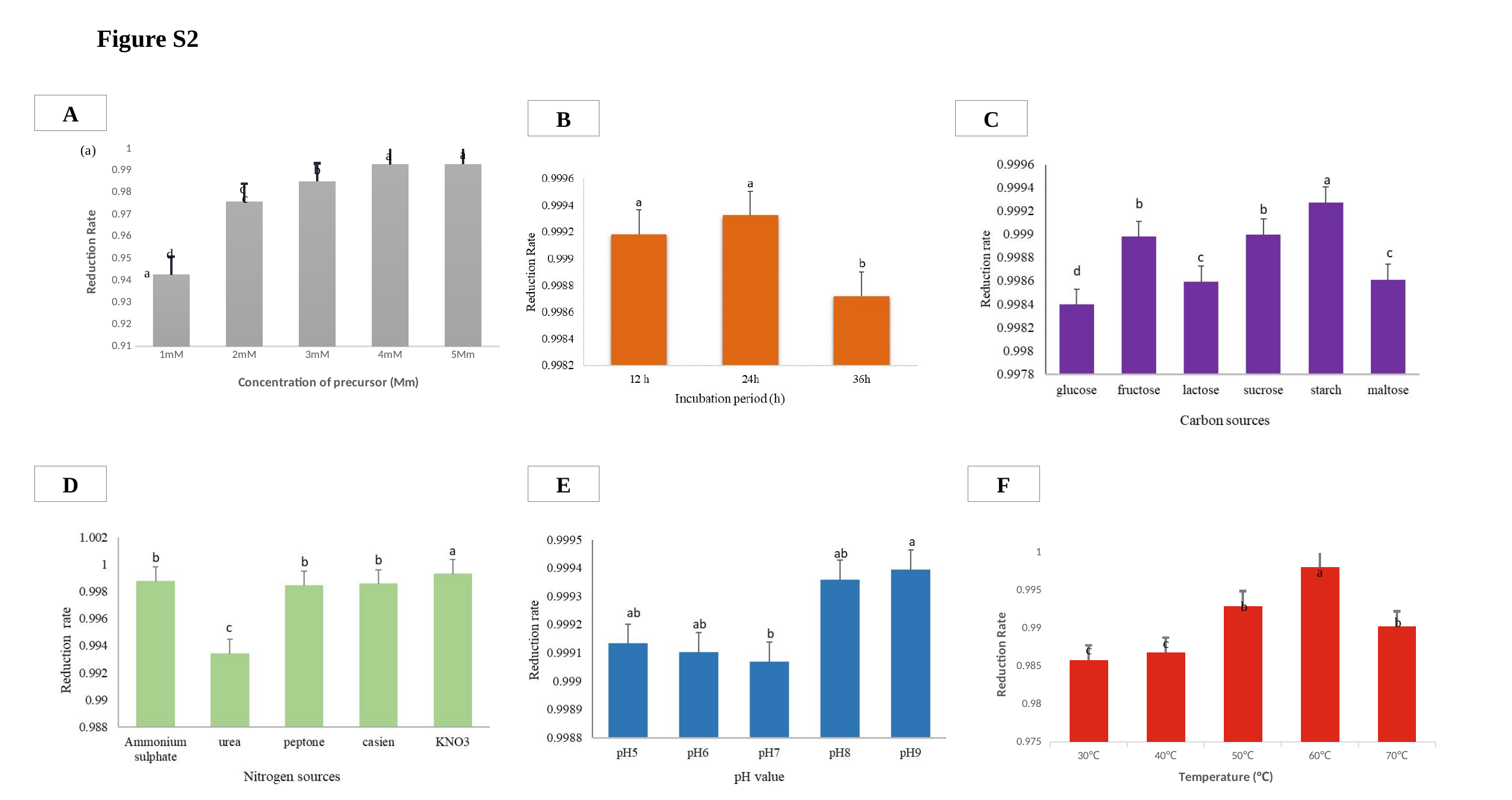

Figure S2
A
B
C
### Chart
| Category | |
|---|---|
| 1mM | 0.942563619 |
| 2mM | 0.975773358 |
| 3mM | 0.985086324 |
| 4mM | 0.992888915 |
| 5Mm | 0.99296996 |
D
E
F
### Chart
| Category | R.R |
|---|---|
| 30℃ | 0.9858041810704391 |
| 40℃ | 0.9868519998257871 |
| 50℃ | 0.9929699603048702 |
| 60℃ | 0.9981184533230802 |
| 70℃ | 0.9903047393101209 |

## Slide 3
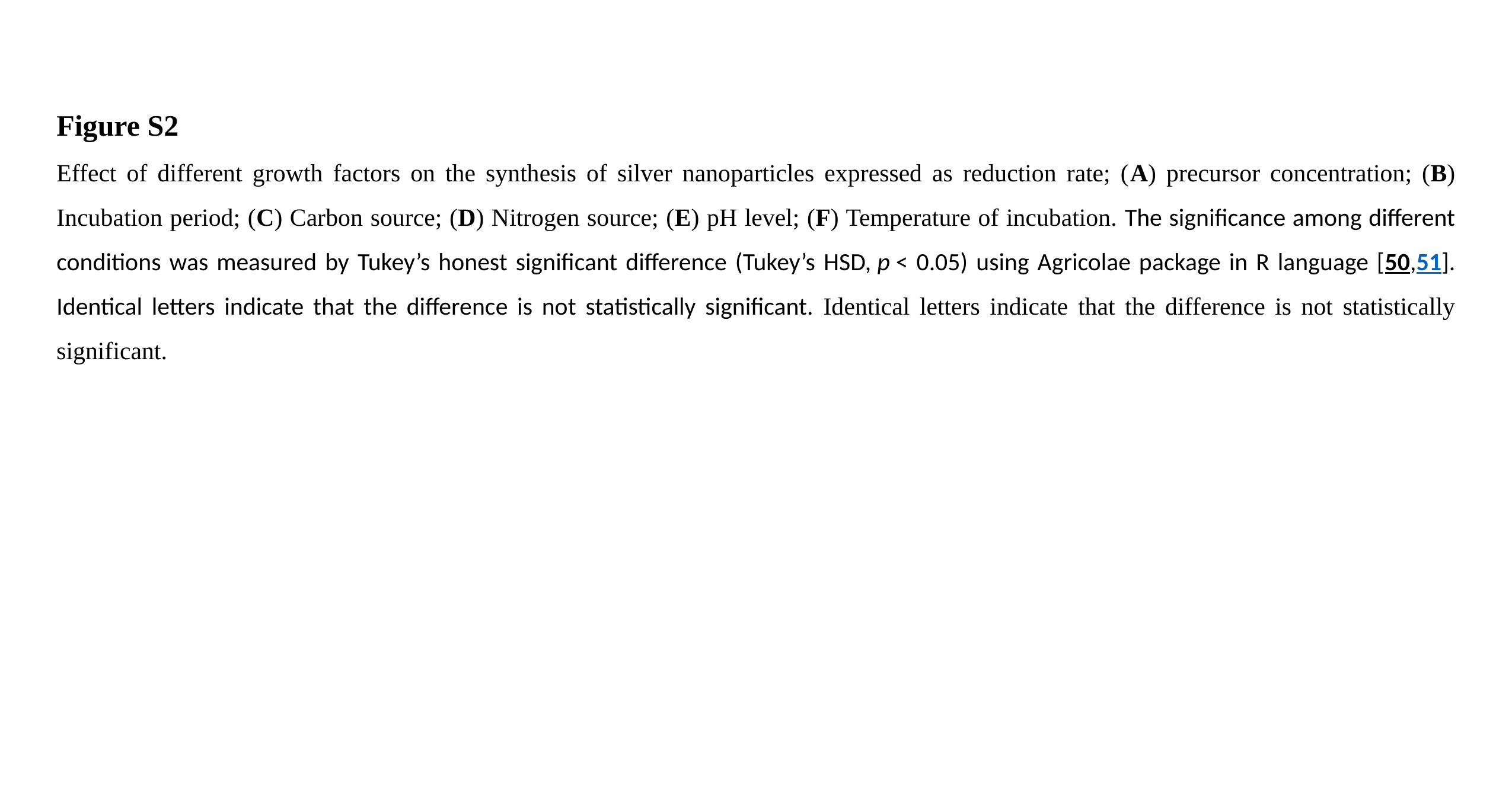

Figure S2
Effect of different growth factors on the synthesis of silver nanoparticles expressed as reduction rate; (A) precursor concentration; (B) Incubation period; (C) Carbon source; (D) Nitrogen source; (E) pH level; (F) Temperature of incubation. The significance among different conditions was measured by Tukey’s honest significant difference (Tukey’s HSD, p < 0.05) using Agricolae package in R language [50,51]. Identical letters indicate that the difference is not statistically significant. Identical letters indicate that the difference is not statistically significant.

## Slide 4
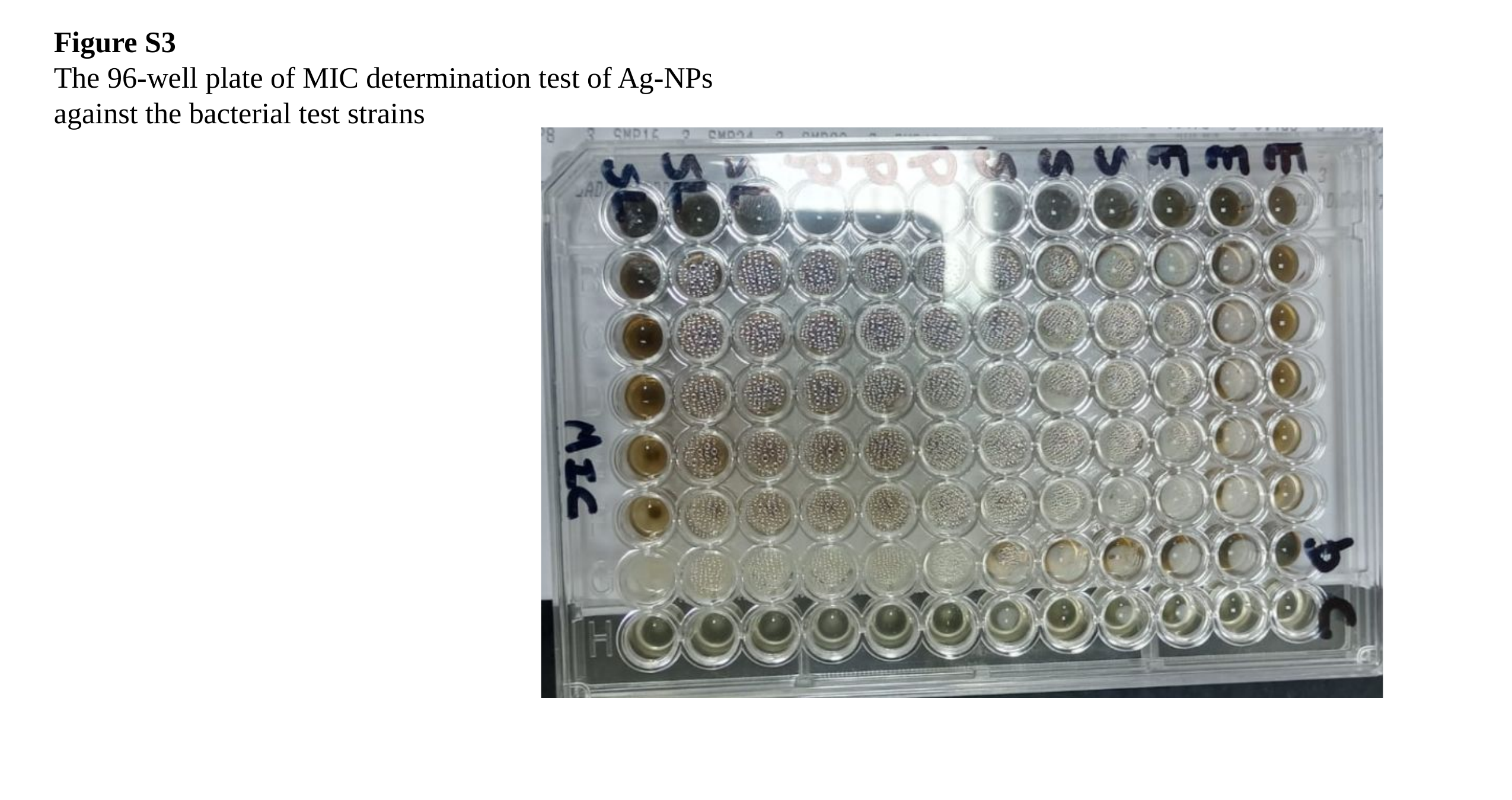

Figure S3
The 96-well plate of MIC determination test of Ag-NPs against the bacterial test strains

## Slide 5
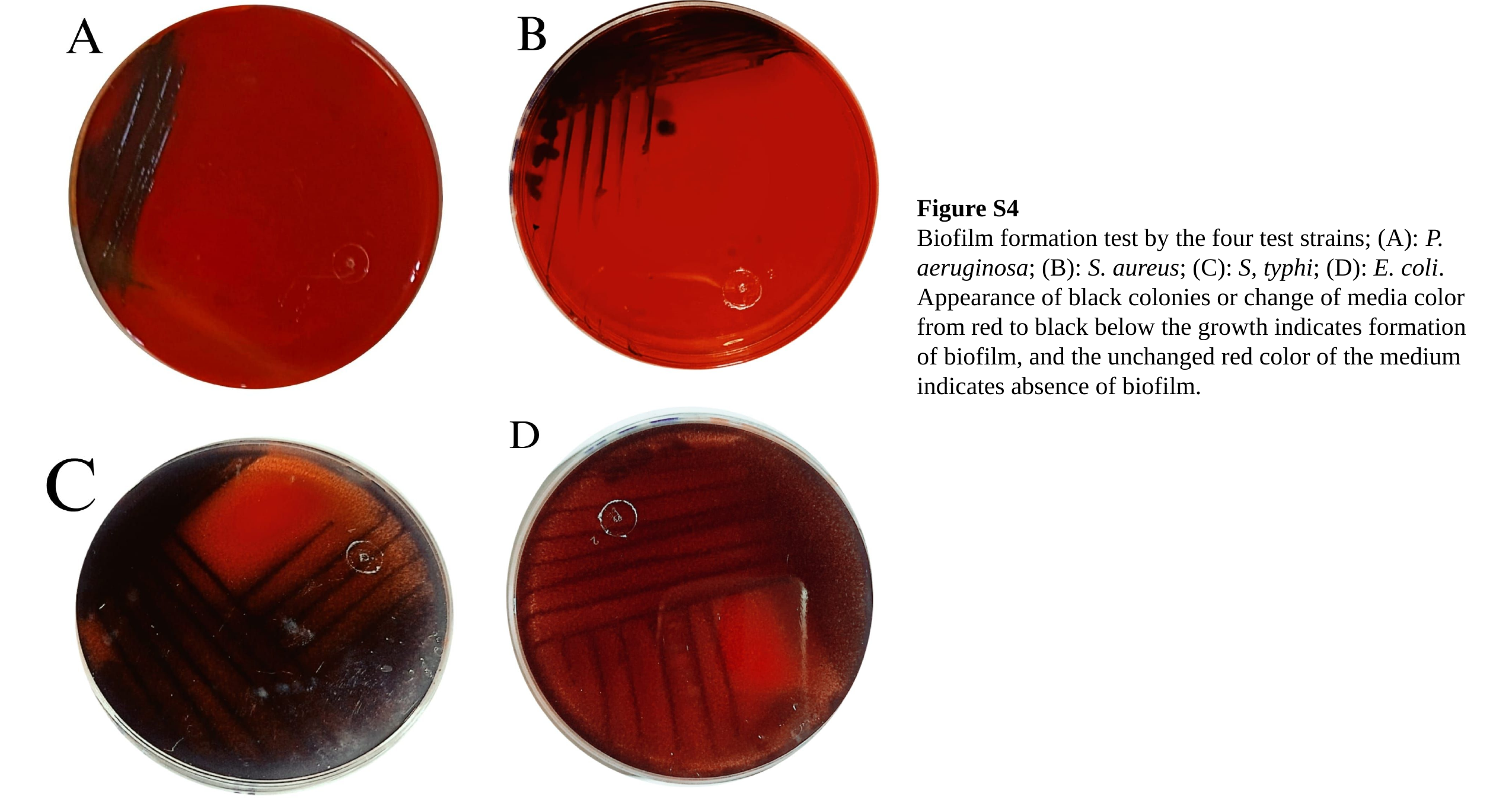

A
Figure S4
Biofilm formation test by the four test strains; (A): P. aeruginosa; (B): S. aureus; (C): S, typhi; (D): E. coli. Appearance of black colonies or change of media color from red to black below the growth indicates formation of biofilm, and the unchanged red color of the medium indicates absence of biofilm.

## Slide 6
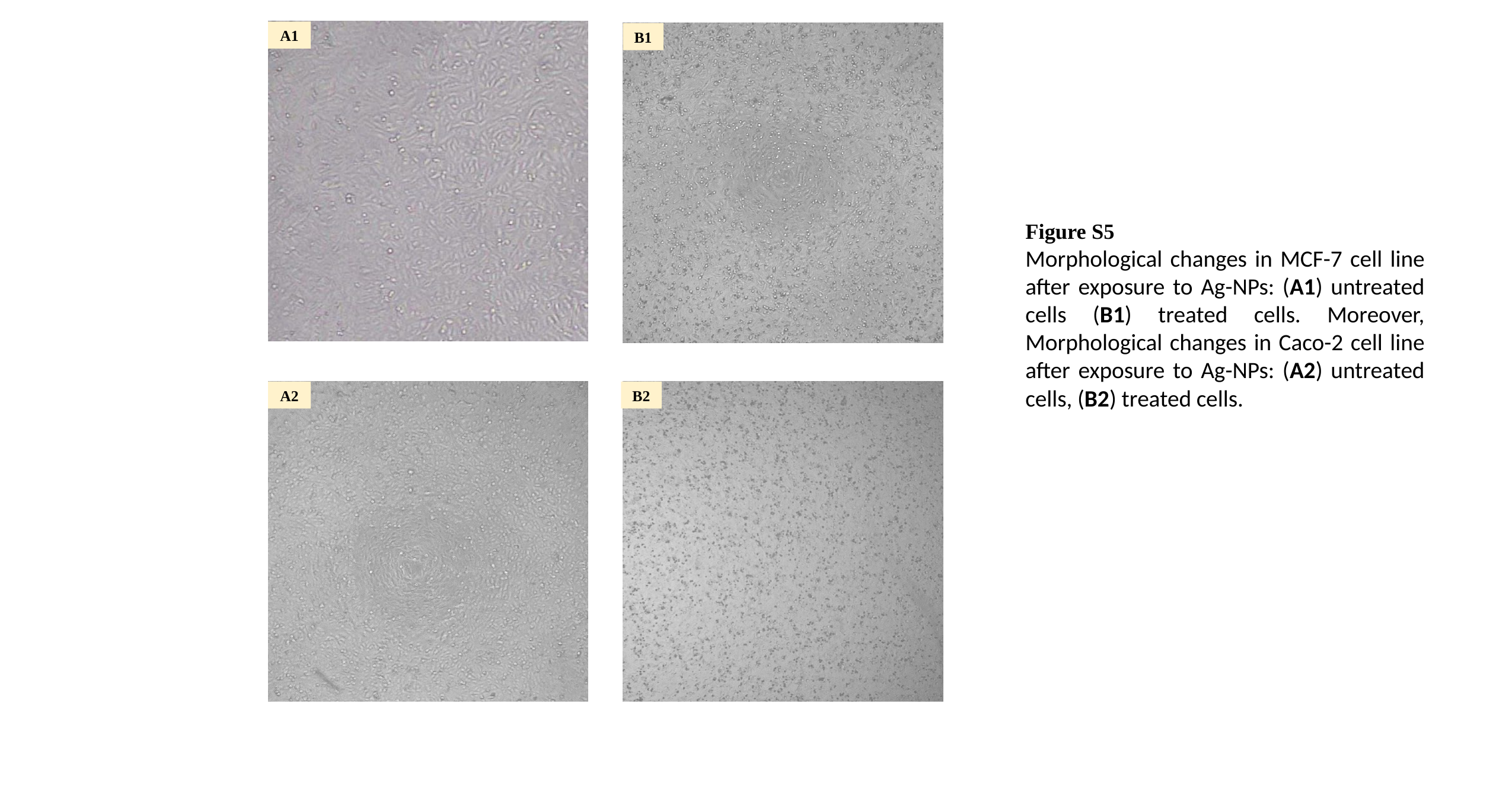

A1
B1
Figure S5
Morphological changes in MCF-7 cell line after exposure to Ag-NPs: (A1) untreated cells (B1) treated cells. Moreover, Morphological changes in Caco-2 cell line after exposure to Ag-NPs: (A2) untreated cells, (B2) treated cells.
A2
B2
